# Zebrafish (*Danio rerio*) larva as an in vivo vertebrate model to study renal function

**DOI:** 10.1152/ajprenal.00375.2021

**Published:** 2022-01-17

**Authors:** Jan Stephan Bolten, Anna Pratsinis, Claudio Luca Alter, Gert Fricker, Jörg Huwyler

**Affiliations:** ^1^Department of Pharmaceutical Sciences, University of Basel, Basel, Switzerland; ^2^Institute of Pharmacy and Molecular Biotechnology, University of Heidelberg, Heidelberg, Germany; ^3^Mount Desert Island Biological Laboratory, Salsbury Cove, Bar Harbor, Maine

**Keywords:** drug transporter, glomerular filtration, kidney tubule, renal function, zebrafish

## Abstract

There is an increasing interest in using zebrafish (*Danio rerio*) larva as a vertebrate screening model to study drug disposition. As the pronephric kidney of zebrafish larvae shares high similarity with the anatomy of nephrons in higher vertebrates including humans, we explored in this study whether 3- to 4-day-old zebrafish larvae have a fully functional pronephron. Intravenous injection of fluorescent polyethylene glycol and dextran derivatives of different molecular weight revealed a cutoff of 4.4–7.6 nm in hydrodynamic diameter for passive glomerular filtration, which is in agreement with corresponding values in rodents and humans. Distal tubular reabsorption of a FITC-folate conjugate, covalently modified with PEG_2000_, via folate receptor 1 was shown. Transport experiments of fluorescent substrates were assessed in the presence and absence of specific inhibitors in the blood systems. Thereby, functional expression in the proximal tubule of organic anion transporter oat (slc22) multidrug resistance-associated protein mrp1 (abcc1), mrp2 (abcc2), mrp4 (abcc4), and zebrafish larva p-glycoprotein analog abcb4 was shown. In addition, nonrenal clearance of fluorescent substrates and plasma protein binding characteristics were assessed in vivo. The results of transporter experiments were confirmed by extrapolation to ex vivo experiments in killifish (*Fundulus heteroclitus*) proximal kidney tubules. We conclude that the zebrafish larva has a fully functional pronephron at 96 h postfertilization and is therefore an attractive translational vertebrate screening model to bridge the gap between cell culture-based test systems and pharmacokinetic experiments in higher vertebrates.

**NEW & NOTEWORTHY** The study of renal function remains a challenge. In vitro cell-based assays are approved to study, e.g., ABC/SLC-mediated drug transport but do not cover other renal functions such as glomerular filtration. Here, in vivo studies combined with in vitro assays are needed, which are time consuming and expensive. In view of these limitations, our proof-of-concept study demonstrates that the zebrafish larva is a translational in vivo test model that allows for mechanistic investigations to study renal function.

## Graphical abstract

**Figure:**
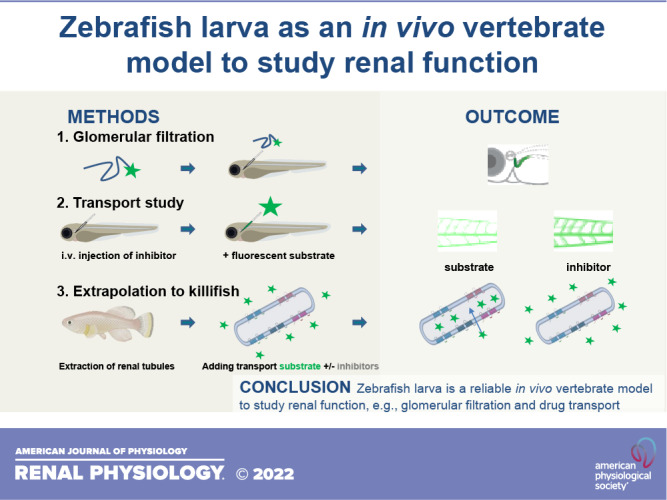

## INTRODUCTION

The kidney is an important excretory organ. The functional unit of the human kidney is the nephron, where blood enters the glomerulus and is passively filtrated. The ultrafiltrate is processed within the proximal convoluted tubule (PCT). Here, small molecules such as salts, water, glucose, citrate, and amino acids are exchanged or recovered to sustain homeostasis. Besides this, epithelial cells of the PCT are equipped with energy-dependent transporters, enabling the active secretion of xenobiotics into the tubular lumen. These drug transporters belong to the superfamilies of ATP-binding cassette (ABC) transporters and solute carriers (SLC), which are either apically or basolaterally expressed (1). Examples include organic anion transporters OAT1–3 (SLC22A66-8) for basolaterally expressed transporters and multidrug resistance gene MDR1/P-glycoprotein (ABCB1), multidrug resistance protein MRP2/4 (ABCC2/4), and breast cancer resistance protein BCRP (ABCG2) for apically expressed transporters (2). Further downstream within the tubules, Na^+^ and K^+^ are processed and vitamins such as folate (FA) are reabsorbed by endocytotic receptor-mediated transport processes (3, 4).

Aquatic animals such as teleosts have highly conserved kidney structures, which are homologous to human. This is not surprising since 70% of protein coding genes of zebrafish have a related counterpart in humans (5). With respect to kidney transporters, ABC-like transporters are phylogenetically conserved in teleosts and higher vertebrates (2). For example, mRNA expression of human homolog renal secretion and reabsorption transporters abcc1, abcc2, and FA receptor 1 (folr1) between 24 and 120 h postfertilization (hpf) was detected in various organs of zebrafish larvae (ZFL), including proximal tubule parts (3, 6–8). Furthermore, abcb4 mRNA expression (a homolog of the human MDR1-like transporter) has been previously described (9). This transporter is involved in the secretion of lipophilic and uncharged xenobiotics. Na^+^-K^+^-ATPase is highly expressed in proximal tubules of zebrafish (*Danio rerio*) and other teleosts, e.g., killifish (*Fundulus heteroclitus*) (2, 10).

The in vivo study of renal function remains a challenge. Such studies are time consuming and expensive and often rely on in vivo experimentation with higher vertebrates. They are often combined with in vitro or ex vivo transporter assays to provide mechanistic insights at a cellular level. The latter models are reliable and provide a high throughput, but a translation to the in vivo situation remains cumbersome (11). In view of these limitations, there is a high unmet need for cost-effective in vivo test systems, which should provide a decent throughput and can be used to bridge the gap between in vitro cell-based assays and in vivo animal experiments in higher vertebrates, including humans. To this end, freshly isolated proximal tubules from killifish can be surgically extracted. They form fully functional sealed tubules when placed in culture media (12, 13). By incubating them with a fluorescent transporter substrate in the presence or absence of specific inhibitors, luminal-directed transport of the fluorescent substrate can be visualized ex vivo by confocal microscopy.

Since this ex vivo model does not respect all relevant kidney functions and aspects of a living organism, in vivo vertebrate models are desired. ZFL can be used for this purpose. They are transparent, undergo fast embryogenesis in that they already hatch by 3 days postfertilization (dpf), are easy to handle, and are therefore frequently used for toxicological, pharmacological, and developmental studies (14). Recent studies have demonstrated that ZFL between 2 and 4 dpf can be used to study the systemic circulation and extravasation of intravenously administered drug formulations (15, 16). These results could be extrapolated to higher vertebrates (i.e., rodents) and establish ZFL as a promising screening model for nanomedicines (17).

Therefore, the aim of this work was to implement ZFL as a translational in vivo vertebrate screening model to study renal function. In contrast to the established ex vivo killifish model mentioned above, the zebrafish model is expected to cover not only proximal tubular secretion but also glomerular filtration and distal tubular reuptake. These processes were studied in this work using fluorescent model compounds including polymers of different molecular weight and substrates of specific transporters in combination with their corresponding inhibitors. Fluorescent test compound distribution within the pronephros and blood system was assessed by confocal microscopy in living ZFL, using recombinant zebrafish lines expressing the fluorescent proteins enhanced green fluorescent protein (eGFP) or mCherry in the endothelium or glomerulus/proximal tubular cells. Finally, tubular secretion of fluorescent substrates was evaluated ex vivo in killifish to compare our results to a well-established tubular secretion model.

## MATERIALS AND METHODS

### Materials

Sulforhodamine 101 (Sulfo101) was purchased from Chemodex (St. Gallen, Switzerland), 8-(2-[fluoresceinyl]aminoethylthio)adenosine-3′,5′-cyclic monophosphate (8-fluo-cAMP) was purchased from Biolog Life Science (Bremen, Germany), a fluorescent cyclosporine A derivative [*N*-ϵ(4-nitrobenzofurazan-7-yl)-D-Lys^8^]cyclosporine A (NBD-CsA) was synthesized as previously described (18), FA-polyethylene glycol PEG-2kDa-FITC was purchased from Biochempeg (Watertown, MA), and NHS-PEG_5_-Mal and NHS-PEG_40_-Mal were obtained from NOF (Tokyo, Japan). SAMSA fluorescein and NHS-TRITC were purchased from Thermo Fisher Scientific (Waltham, MA). Agarose, probenecid, erythromycin, verapamil hydrochloride, *p*-aminohippuric acid, MK-571 sodium salt hydrate, zosuquidar hydrochloride, saquinavir, indinavir sulfate salt hydrate, folic acid, fluorescein sodium salt (Fluo), FITC-dextran (40, 70, or 150 kDa), 1-phenyl-2-thiourea (PTU), and ethyl-3-aminobenzoate methanesulfonate (MS-222, tricaine) were purchased from Sigma-Aldrich (Buchs, Switzerland).

### Ethical Approval

All animal experiments were carried out in accordance with local animal welfare regulations.

### LogD_7.4_ Prediction

The logD_7.4_ of test compounds was predicted using the Partitioning PlugIn of Marvin Sketch 20.19.0 software (ChemAxon Europe, Budapest, Hungary).

### Fluorescent Labeling of PEG_5_ and PEG_40_

Coupling of SAMSA fluorescein was performed according to the manufacturer’s instructions. In brief, SAMSA fluorescein was incubated in 100 mM NaOH for 10 min before being added to NHS-PEG_5_ or NHS-PEG_40_-Mal at a two- to threefold excess in 100 mM NaPi, 150 mM NaCl, and 5 mM EDTA (pH 7.2). The SAMSA fluorescein PEG mixtures were left to react overnight at room temperature, before purification by gel filtration (Sephadex G50 fine), as previously described (19). Collected fractions were pooled and concentrated using 3-kDa cutoff Amicon filter device (Merck Millipore, Burlington, MA).

### Fluorescence Correlation Spectroscopy

The hydrodynamic diameter (*D*_H_) of fluorescent-labeled polymers [i.e., FITC-dextran (40, 70, or 150 kDa) and FITC-PEG (5 and 40 kDa)] was determined by fluorescence correlation spectroscopy. An Olympus IX73 inverted microscope (Olympus, Tokyo, Japan) using an immersion Super Apochromat objective (1.2 numerical aperture, ×60, UplanSApo, Olympus) was used to perform the measurements. Emitted photons were filtered with a bandpass filter (512 nm) before detection with a single-photon avalanche diode (SPCM CD3516H, Excelitas). The free dye Atto 488 carboxylic acid (*D* = 400 μm/s^2^ at 298 K, Thermo Fisher Scientific) was dissolved at a concentration of 10 nM in double distilled water and used to calibrate the confocal volume of the excitation channel at 481 nm. Intensity fluctuations were recorded over 60 s. The experimental autocorrelation curves of the calibration dye Atto 488 and the fluorophore coupled samples were fitted with a one-component triplet state model, as previously described (20). Data were processed using PicoQuant Software (Berlin, Germany).

### Intravenous Injections and Imaging of ZFL

Animal experiments and husbandry were carried out in accordance with Swiss animal welfare regulations. Regarding the terminology of zebrafish, we followed the life stage definitions established by Kimmel et al. (21), who defined >72 hpf old zebrafish as “larvae.” Eggs from adult zebrafish (Table 1) were collected from different parents at 0.5–1 hpf and kept at 28°C in zebrafish culture media (25).

**Table 1.** Transgenic zebrafish lines with the corresponding promoter coupled to either eGFP or mCherry indicating which organ is fluorescently marked

Transgenic Line	Characteristics	Reference	Source
AB/Tübingen	Wild-type		Prof. Dr. Affolter, Basel, Switzerland
Tg(wt1b:eGFP)	Glomerulus and proximal convoluted tubule GFP marker	(22)	Prof. Dr. Schiffer, Erlangen, Germany
Tg(kdrl:eGFP)	Endothelium GFP marker	(23)	Prof. Dr. Affolter, Basel, Switzerland
Tg(kdrl:mcherry-CAAX)	Endothelium mCherry marker	(24)	Prof. Dr. Affolter, Basel, Switzerland

Shown is an overview of the transgenic lines. eGFP, enhanced green fluorescent protein.

The number of larvae in a 25-mL dish did not exceed 100. The formation of pigment cells was suppressed by adding 30 μg/mL PTU to the media. Then, 72 and 96 hpf hatched ZFL were embedded in 0.3% agar containing PTU and tricaine (0.01%). Experiments were carried out at room temperature. Randomly chosen larvae were injected with a calibrated volume of 1–2 nL of 0.1–2 mM stock solutions of test compounds into the cardinal vein (CV) above the heart. Embryos were obtained from the mating of six male and six female adult zebrafish and were randomly assigned to the treatment groups. Water-soluble substances were dissolved in PBS. Lipophilic substances such as NBD-CsA, erythromycin, and probenecid were administered in an up to 15% (vol/vol) DMSO-PBS solution by injection of 2 × 1 nL with a delay of 30 s between injections. FA-PEG_2000_-FITC was dissolved in Tris buffer (pH 9.3). Chemically reactive compounds (i.e., Sulfo101) were incubated for 2.5 h in FCS (BioConcept Amimed, Allschwil, Switzerland) to neutralize reactive moieties followed by a short centrifugation before injection. For intravenous injections, a micromanipulator (Wagner Instrumentenbau, Schöffengrund, Germany), a pneumatic Pico Pump PV830 (World Precision Instruments, Sarasota, FL), and a Leica SAPO microscope (Leica, Wetzler, Germany) were used. Tail regions were imaged 0.5–9 h postinjection (hpi) using an Olympus FV3000 confocal laser scanning microscope equipped with a ×20 UPIabSApo (numerical aperture of 0.75) objective and a ×30 UPIanSApo (numerical aperture of 1.05) objective. Confocal images were acquired using a sequential line scan, excitation wavelengths of 488 and 561 nm (argon laser), and emission wavelengths of 500–540 and 570–620 nm, respectively. Of note, no signal cross talk was detected between the different channels.

To reduce intraexperimental variability, comparative experiments were performed during the same day by the same operator and using the same stage. Droplet size consistency (sample volume) was permanently checked using a reticle mounted on the eyepiece of a Leica SAPO binocular to ensure that the same volume of compound was injected throughout the course of the experiment. Reproducibility was verified by repeating series of experiments on at least three different days. For each condition of each series of experiments at least five zebrafish larvae were used (*n* = 5). For the preparation of figures and the corresponding quantitative analysis, data from one representative series of experiments were used.

### Signal Intensity Quantification and Postprocessing of Images

Obtained confocal microscopy images were analyzed and edited using OMERO software 5.4.10 (https://www.openmicroscopy.org/omero/) as an image processing program. Quantitative signal intensities in distinct organs were evaluated using Fiji software 2.1.0/1.53c (https://imagej.net/software/fiji/) and were done as follows: for drug transport experiments (i.e., tubular secretion and endocytosis), zebrafish lines were used, which express a fluorescent marker (i.e., kdrl:eGFP or kdrl:mCherry or wt1b:eGFP) in vascular endothelia or renal epithelia. This allowed for a localization of fluorescent-labeled compounds within a defined three-dimensional anatomic structure such as the dorsal artery (DA) or kidney tubule. Signals were quantified by measuring mean signal intensities within this selected region of interest. Untreated controls and all corresponding treatment groups were analyzed using the same laser and microscopy settings, which allowed for a direct comparison of treatment groups. Alternatively (Figs. 2 and 7), regions of interest were selected based on bright-field microscopy images. Analysis of the DA was used to quantify signals of circulating fluorophores within the blood compartment. Quantification of signal intensities was based on maximum intensity projections. Signal intensities are presented as fold changes normalized to the mean of the (experimental) control.

### Killifish and Tissue Preparation

Killifish (*F. heteroclitus*) proximal kidney tubules were isolated and prepared as previously described (12). In brief, killifish were purchased from local fishermen in the vicinity of Mount Desert Island, ME and maintained at the Mount Desert Biological Laboratory in tanks with natural flowing, aerated sea water. Since no sex-related differences were observed, extracted tubules from at least six randomly chosen killifish were incubated in enriched marine teleost buffer (140 mM NaCl, 2.5 mM KCl, 1.5 mM CaCl_2_, 1 mM MgCl_2_, and 20 mM Tris) containing 1 μM of the indicated fluorescent substrate in the presence or absence of a 10- to 20-fold excess of a nonfluorescent inhibitor. Incubations were carried out at 8°C. Tubular accumulation of fluorescence signals was monitored using an Olympus FV1000 inverted confocal laser scanning microscope (×20, PlanFluo Dry, numerical aperture 0.5). Signal intensities were quantified using image capture and analysis software (NIH Image 1.61, https://imagej.nih.gov/).

### Statistical Analysis

Statistical analysis was performed with GraphPad Prism v. 8.0.2 (GraphPad Software, San Diego, CA) using unpaired two-tailed *t* test analysis for direct comparisons. Where appropriate, individual data points are presented as dot plots next to the average and SD for the group.

## RESULTS

**Figure 1.**
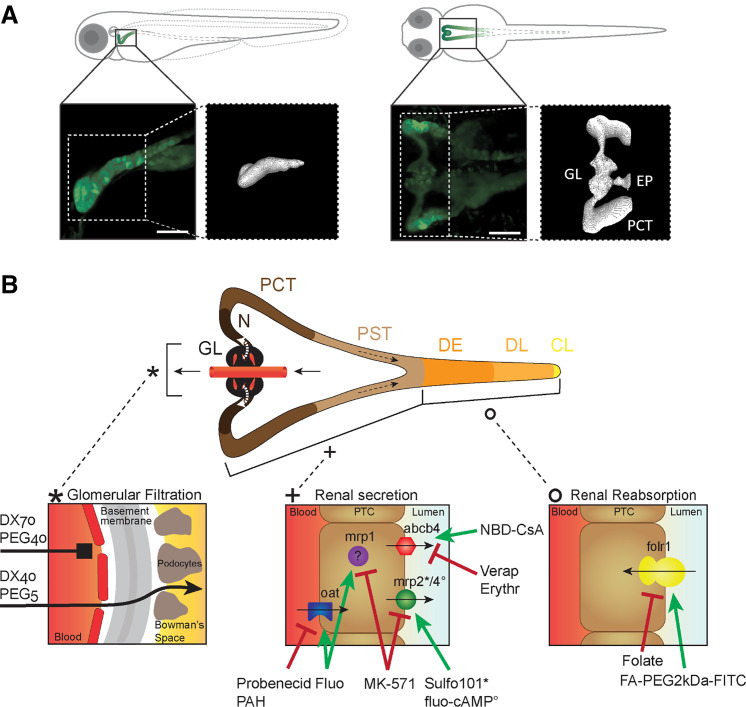
Anatomic localization of the pronephros in a 72-h postfertilization (hpf) zebrafish larva (ZFL) and schematic representation of its functional units. *A*: lateral and ventral projection of a 72-hpf tg(wt1b:eGFP) ZFL expressing enhanced green fluorescent protein (eGFP) mainly in the proximal convoluted tubule (PCT) and glomerulus (GL). A faint signal was also present in the exocrine pancreas (EP). A three-dimensional projection of the pronephros is shown. Scale bars = 50 µm. *B*: the pronephros consists of two nephrons with a fused GL, neck (N), PCT, proximal straight tubule (PST), distal early (DE), late distal (DL), and collecting duct and cloaca (CL). Renal function encompasses glomerular filtration (*left*), renal secretion (*middle*), and renal reabsorption (*right*). Transporters are listed together with their substrates (green label) and inhibitors (red label) used in this study. abcb4, zebrafish homolog of human MDR1; DX, dextran; Erythr, erythromycin; FA, folate; Fluo, fluorescein sodium salt; folr1, folate receptor 1; mrp, multidrug resistance-associated protein; NBD-CsA, NBD-labeled cyclosporin A; oat, organic anion transporter; PAH, *p*-aminohippurate; PEG, polyethylene glycol; PTC, proximal tubule cell; Sulfo101, sulforhodamine 101; Verap, verapamil hydrochloride.

The pronephros of ZFL consists of two nephrons that are combined at the larva’s midline just ventral to the dorsal aorta (26). For a better anatomic visualization of the pronephros, genetically modified tg(wt1b:eGFP) ZFL, expressing eGFP in epithelial cells of the PCT and glomerulus, were used. In Fig. 1*A*, a confocal image of this triangle-shaped organ can be seen in a lateral and ventral projection. Figure 1*B* shows a schematic representation of three distinct sections of the ZFL pronephros indicating the three main kidney processes, passive glomerular filtration, active proximal tubular excretion, and active distal tubular reabsorption. Examples of the transport proteins of interest are provided, and their expression at defined membrane domains (basolateral or luminal) of epithelial cells is shown. Thus, the used fluorescent model compounds including polymers of different molecular weight and substrates of specific transporters in combination with their corresponding inhibitors are illustrated.

**Figure 2.**
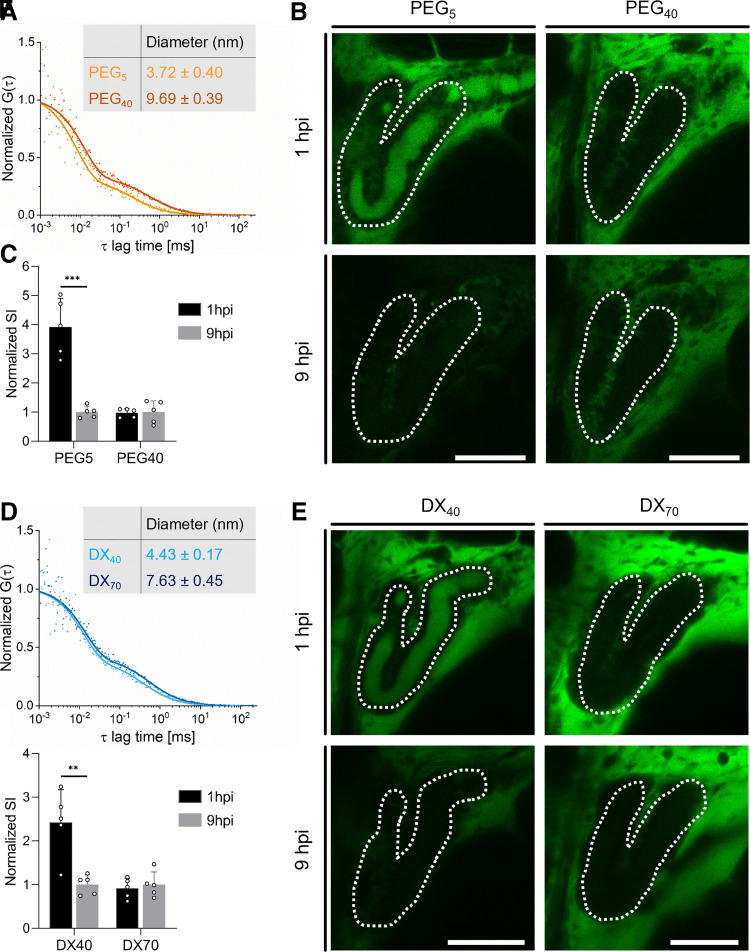
Qualitative and quantitative assessment of glomerular filtration in 96-h postfertilization (hpf) zebrafish larvae (ZFL). *A*: the hydrodynamic diameter of polyethylene glycol (PEG) with a molecular weight of 5 and 40 kDa was determined by fluorescent correlation spectroscopy. The autocorrelation function G(τ) plotted over lag time τ was used to calculate the hydrodynamic diameter of PEGs. *B*: proximal convoluted tubules (PCTs) are marked with white dotted lines. Shown is a qualitative assessment of PEG (green signal) within the proximal tubular lumen [5- and 40-kDa PEG, 1 or 9 h postinjection (hpi)]. *C*: quantitative assessment of luminal PEG signals. Signal intensities were normalized to the respective mean at 9 hpi. *D*–*F*: same experimental setup as in *A–C* using dextrans (DX) with molecular weights of 40 and 70 kDa. Values are means ± SD, *n* = 5. **P < 0.005; ***P < 0.0005. Scale bars = 50 µm.

In a first step, glomerular filtration was investigated. This includes the evaluation of the presence of an adequate barrier to sieve molecules based on their physicochemical properties (e.g., size), whether a molecular weight cutoff can be established, and how this relates to values obtained from literature for higher vertebrates. The glomerular filtration molecular weight cutoff in the 96-hpf wild-type ZFL was determined using fluorescent dextran and PEG conjugates. FITC-DX was commercially available; larger molecular weight PEG was instead coupled to SAMSA fluorescein in house. The *D*_H_ and purity of these polymers were measured using fluorescence correlation spectroscopy. We obtained a diffusion coefficient of 125 and 48 μm^2^/s for FITC-PEG_5_ and FITC-PEG_40_ samples, respectively. This corresponds to a *D*_H_ of 3.7 and 9.7 nm (Fig. 2*A*). For FITC-DX_40_ and FITC-DX_70_, diffusion coefficients of *D* = 105 and 61 μm^2^/s translate to a *D*_H_ = 4.4 and 7.6 nm, respectively (Fig. 2*D*). Their glomerular filtration in vivo, assessed by detection of cleared substances in the lumen of the PCT, was determined at 1 and 9 hpi by confocal microscopy. A representative confocal image of the pronephros is shown in Fig. 2, *B* and *E*. For quantitative analysis, the luminal signals were each normalized to 9 hpi and are shown in Fig. 2, *C* and *F*. Qualitative as well as quantitative analysis revealed the accumulation of small-molecular weight PEG and dextran within the proximal tubular lumen, being indicative of glomerular filtration, whereas high-molecular weight polymers were not filtered. The cutoff of glomerular filtration in ZFL corresponds to a *D*_H_ of the polymers between 4.4 nm and 7.6 nm. The *D*_H_ for 40-kDa PEG is significantly higher than similar 40-kDa dextran polymers, indicating that not the molecular weight but the *D*_H_ determines the cutoff of glomerular filtration.

**Figure 3.**
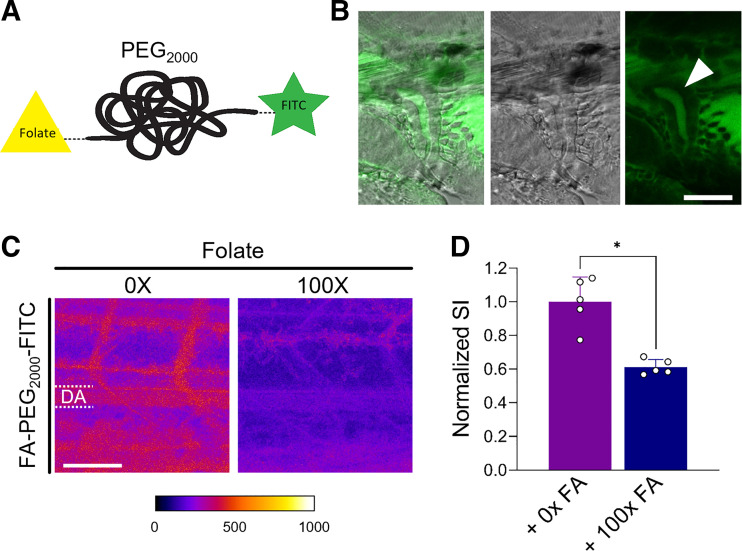
Reabsorption of folate (FA) in the distal tubule. *A*: FA receptor 1 (folr1)-mediated distal tubular reabsorption was studied using a FA conjugate covalently modified with polyethylene glycol (PEG; molecular weight: 2,000 Da) and the fluorescent dye FITC. *B*: accumulation of in the lumen of the distal tubule 5 min postinjection of a 72-hpf zebrafish larva (ZFL). Scale bar = 30 µm. *C*: confocal microscopy image of the tail region of a 72-hpf ZFL 1 h after intravenous injection of a fluorescent-labeled FA-PEG_2000_-FITC derivative in the presence and absence of a 100-fold excess of native FA (100× FA). *D*: quantitative evaluation of the dorsal artery (DA) in *C*. Signal intensities (SI) were normalized to the mean of the control (no inhibitor, 0× FA). Values are means ± SD; *n* = 5. *P < 0.0001. Scale bar = 50 µm.

Next, distal tubular reuptake of FA by the folr1 homolog transporter was investigated using a fluorescent labeled derivative of FA, i.e., FA-PEG_2000_-FITC (Fig. 3*A*). The purity and *D*_H_ of the fluorescent polymer were characterized using fluorescence correlation spectroscopy. The *D*_H_ of the FA-PEG_2000_-FITC conjugate was 1.08 ± 0.57 nm. The filtered fluorescent FA-PEG polymer appeared in the lumen of the distal tubule 5 min after injection of a high concentration (1 nL of a 2 mM solution). These high concentrations were necessary to allow for detection in the patent luminal space of the distal tubule. To assess FA-mediated tubular reabsorption, a 100-fold molar excess of native FA as a specific inhibitor was preinjected 10 min before administration of 1 nL of 0.1 mM FA-PEG_2000_-FITC. Intensity within the DA was assessed 1 hpi to evaluate the extent of reabsorption from the tubular lumen back into the bloodstream. A representative confocal image is shown in Fig. 3*C*, *left*, demonstrating higher signal intensities (red > blue) in the DA compared with FA-preinjected ZFL. This is indicative of continuous tubular reuptake of FA-PEG_2000_-FITC in ZFL. This reuptake is reduced by a factor of 2 in the presence of an excess of native FA, which indicates competitive tubular reabsorption via folr1 (Fig. 3*D*, *right*).

Proximal tubular secretion was investigated based on specific clearance of fluorescent labeled transporter substrates from the bloodstream. The transport substrate/inhibitor pairs selected for this study were selected based on a comprehensive review of the existing literature. Table 2 thus shows information on the ABC- and SLC-transporter specificity of fluorescent substrates and nonfluorescent inhibitors in different teleost species and their tissue-specific expression patterns. The specificity of fluorescent substrates and nonfluorescent inhibitor is shown in Table 2.

**Table 2.** Overview of organ specific drug transporters in different teleost species

Transporter	Species	Organ	Substrate	Inhibitor	References
MDR1-like (ABCB1-like)	Killifish (*Fundulus heteroclitus*)	Isolated renal proximal tubules	NBD-CsA	Rapamycin^1^, octreotide^2^, ivermectin^3^, PSC-833^4^, S1P^4^, FTY^4^, CsA^5^, CsG^5^, verapamil^5^, vinblastine^5^, DNP^5^, KCN^5^, Quin^5^	(27)^1^, (28)^2^, (12, 18)^3^, (29)^4^, (18)^5^
			NBD-rapamycin	Rapamycin, CsA, verapamil, FK506, PSC-833	(27)
			BODIPY-ivermectin	PSC-833, verapamil	(12)
			BODIPY-verapamil	PSC-833, S1P	(29)
			NBD-octreotide	Octreotide, verapamil, CSA, PSC-833, LTC4	(28)
		Isolated brain capillaries	NBD-CsA	PSC-833, CsA	(30)
			BODIPY-verapamil	PSC-833	(30)
	Dogfish (*Squalus acanthias*)	Isolated brain capillaries	NBD-CsA	CsA, PSC-833	(30)
			BODIPY-verapamil	PSC-833	(30)
			BODIPY-ivermectin	PSC-833	(30)
	Rainbow trout (*Oncorhynchus mykiss*)	Isolated hepatocyctes	Rhodamine 123	Verapamil^1,2^, vinblastine^1^, doxorubicin^1^, CsA^1,2^, VO_3_^1^, vinblastine^1^, reversin 205^2^, MK-571^1,2^	(31)^5^, (32)^2^
			Calcein-AM	Reversin 205, verapamil, CsA, MK-571	(32)
			BODIPY-verapamil	Reversin 205	(32)
OAT1-3-like (SLC226-8-like)	Killifish (*F. heteroclitus*)	Isolated renal proximal tubules	Fluorescein (FL)	PAH^1,2,4,5^, probenecid^1,4^, CdCl_2_^3^, HgCl_2_^3^	(12)^1^, (18)^2^, (33)^3^, (34)^4^, (29)^5^, (35)^6^
	Dogfish (*S. acanthias*)	Isolated choroid plexus	Fluorescein (FL)	(2,4-D), probenecid	(36)
MRP2-like (ABCC2-like)	Killifish (*F. heteroclitus*)	Isolated renal proximal tubules	FL-MTX	LTC4^1,2^, octreotide^1^, ivermectin^2^, CdCl_2_^4,8^, HgCl_2_^4,8^, PAH^5^, probenecid^5^, MTX^5^, folate^5^, BSP^5^, BCG^5^, CsA^5^, verapamil^5^, TEA^5^, MK-571^6^, ET^7,8^, PTH^7^, PTHrP^7^, SNP^8^, PMA^8^, gentamicin^8,9^, amikacin^8^, diatrizoate^8^, 8-BrcGMP^8^, RP-8-BrcGMP^8^	(28)^1^, (12)^2^, (13)^3^, (33)^4^, (34)^5^, (29)^6^, (37)^7^, (38)^8^, (35)^9^
		Isolated brain capillaries	FL-MTX	LTC4	(30)
	Dogfish (*S. acanthias*)	Isolated brain capillaries	FL-MTX	LTC4	(30)
		Isolated choroid plexus	FL-MTX	Probenecid, folate, MTX, taurocholate, PAH, ES, digoxin, LTC_4_, MK-571	(39)
	Killifish (*F. heteroclitus*)	Isolated renal proximal tubules	Sulfo101 (Texas red)	MK-571^1^, CdCl_2_^1^, PAH^2^, probenecid^2^, CsA^2^, verapamil^2^, LTC_4_^2^	(40)^1^, (34)^2^
	Killifish (*F. heteroclitus*)	Isolated brain capillaries	Sulfo101 (Texas red)	LTC_4_	(30)
	Dogfish (*S. acanthias*)	Isolated choroid plexus	Sulfo101 (Texas red)	ES, digoxin, TC, MTX, MK-571	(41)
		Isolated rectal gland tubules	Sulfo101 (Texas red)	ET-1, big ET-1, PMA, forskolin, RP-cAMP	(42)
MRP-like	Zebrafish (*Danio rerio*)	Zebrafish 24 hpf ionocytes	BCECF-AM	PSC-833, CsA, MK-571	(43)
MRP4-like (ABCC4-like)	Killifish (*F. heteroclitus*)	Isolated renal proximal tubules	Fluo-cAMP	MK-571, LTC4, AZT, cAMP, adefovir (PMEA), 8-bromo-cGMP	(13)
BCRP2-like (ABCG2-like)	Killifish (*F. heteroclitus*)	Isolated renal proximal tubules	Mitoxantrone	KO143, FTC	(44)
Abcb4 (ABCB1-like)	Zebrafish (*D. rerio*)	Zebrafish embryo 48 hpf	Rhodamine B	CsA, PSC-833, vinblastine, verapamil, phenanthrene, tonalide, vincristine, doxorubicin, galaxolide	(9)
		Zebrafish 24 hpf ionocytes^2^/48 hpf embryo^1^	Calcein-AM	CsA^1,2^, PSC-833^1,2^, vinblastine^2^	(9)^1^, (43)^2^
		Zebrafish 24 hpf ionocytes	DiOC6(3)	CsA, PSC-833, MK-571, verapamil	(43)

Shown are the used fluorescent model substrates and their corresponding inhibitors. (2,4-D), 2,4-dichlorophenoxyacetic acid; AZT, azidothymidine; BCECF-AM, 2′,7′-bis(2-carboxyethyl)-5(and 6)-carboxyfluorescein-AM; BCG, bromocresol green; BSP, bromosulfophthalein; DiOC6(3), 3,3′-dihexyloxacarbocyanine iodide; DNP, 2,4-dinitrophenol; ES, estrone sulfate; ET-1, endothelin-1; FTC, fumitremorgin; FTY, FTY720 is a prodrug of FTY720P; MTX, methotrexate; PAH, *p*-aminohippurate; PMA, phorbol-12-myristate-13-acetate; PTH, parathyroid hormone; PTHrP, resnPTHrP [sea bream (*Sparus auratur*) recombinant parathyroid hormone-related protein]; quin, quinine; RP-8-BrcGMP, inactive isoform of 8-BrcGMP; RP-cAMP, cAMP analog that does not activate PKA; S1P, sphingosine-1-phosphate; SNP, sodium nitroprusside; TC, taurocholate; TEA, tetraethylammonium; V0_3_, vanadate.

**Figure 4.**
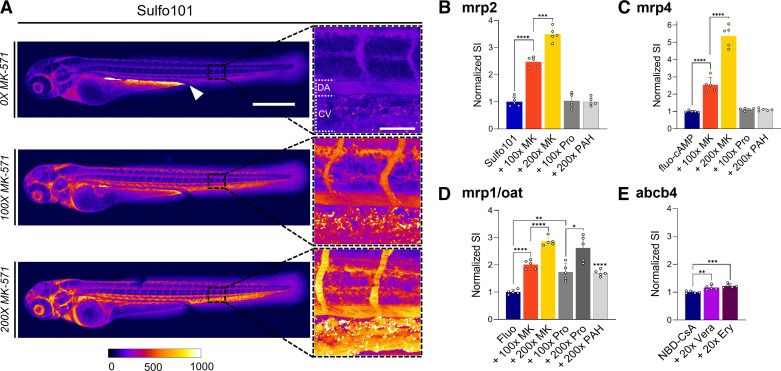
Proximal tubular secretion of fluorescent labeled substrates of drug transporters. *A*: confocal microscopy analysis of 72-h post fertilization (hpf) zebrafish larvae (ZFL) at 1 h postinjection (hpi) of sulforhodamine 101 (Sulfo101) in the presence of increasing concentrations of the multidrug resistance-associated protein (mrp) inhibitor MK-571 (MK). The white arrow shows the gastrointestinal tract and cloaca. Scale bar = 500 µm. Magnified sections of the tail region vasculature are shown. Increasing signal intensity is shown from blue to red to white. Scale bar = 50 µm. *B–E*: quantitative analysis compared with control (no inhibitor) of 72 hpf ZFL injected with the indicated transporter substrate (blue bar) and a *x*-fold excess of inhibitor. The signal was quantified within the dorsal artery (DA). The inhibitors used were as follows: probenecid (Pro), *p*-aminohippurate (PAH), verapamil (Vera), and erythromycin (Ery). Values are means ± SD; *n* = 5. *P < 0.05; **P < 0.005; ***P < 0.0005; ****P < 0.0001. CV, cardinal vein; Fluo, fluorescein; NBD-CsA, NBD-cyclosporine A.

To evaluate tubular secretion, 72–80 hpf tg(kdrl:eGFP/mCherry-CAAX) ZFL were preinjected and incubated with a specific inhibitor (e.g., 10 min except for 1 h for verapamil) followed by the injection of a fluorescently labeled substrate. One hour postinjection, the tail region was imaged and signal intensity within the DA was compared with ZFL that were not preinjected with the corresponding inhibitor. Starting with apically located transporters, the functionality of mrp2 (abcc2) was assessed using Sulfo101 (or Texas red) as a fluorescent substrate and MK-571 as an inhibitor. Inhibitor concentration-dependent inhibition of mrp2 by MK-571 resulted in reduced renal secretion and thus enhanced retention in the bloodstream (Fig. 4*A*). Quantitative analysis of the fluorescent signal within the DA revealed a three- to fourfold increase in plasma concentrations compared with control (i.e., absence of MK-571). Of note, in these experiments, an increase in Sulfo101 secretion was associated with the appearance of a fluorescent signal in the yolk next to the cloaca. This could be indicative of local reabsorption of excreted Sulfo101. As expected, inhibitors of the basolaterally expressed SLC oat (slc22), i.e., probenecid and *p*-aminohippurate, did not interfere with transport of the mrp2 substrate Sulfo101 (Fig. 4*B*). Likewise, transport of the mrp4 (abcc4) substrate fluo-cAMP was inhibited by MK-571 but not by probenecid and *p*-aminohippurate, leading to an up to 5.5-fold increase in plasma concentrations (Fig. 4*C*). Transport of Fluo, a substrate of both ABC transporter mrp1 and SLC oat (slc22), was sensitive to inhibition by MK-571, probenecid, and *p*-aminohippurate (Fig. 4*D*). NBD-CsA, a substrate of the P-glycoprotein analog abcb4 in ZFL, showed a statistically significant sensitivity toward typical inhibitors of human P-glycoprotein (ABCB1), namely, verapamil and erythromycin (Fig. 4*E*).

**Figure 5.**
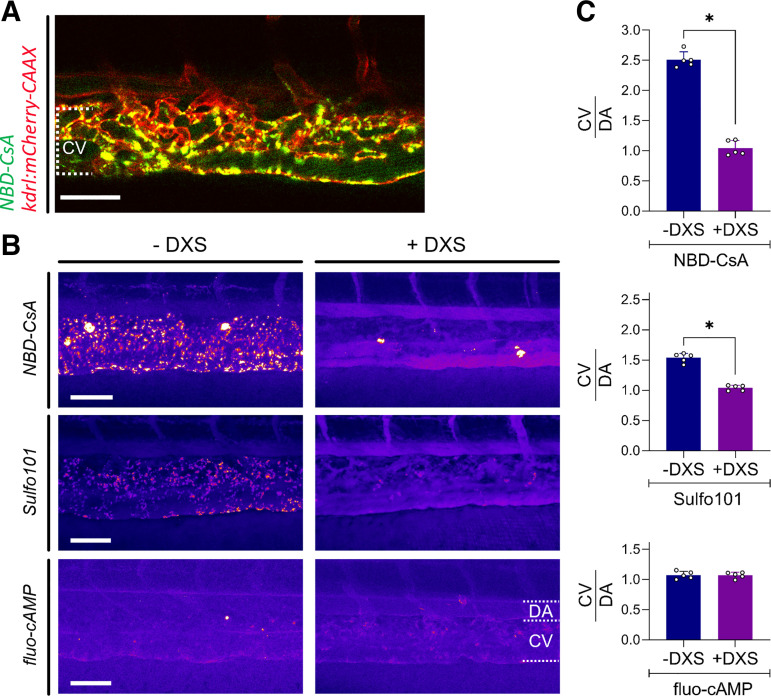
Cellular uptake by scavenger endothelial cells of fluorescent test compounds in 72 h postfertilization (hpf) zebrafish larvae (ZFL). *A*: accumulation of fluorescent NBD-cyclosporine A (NBD-CsA; green signal) 1 h postinfection (hpi) in the dorsal cardinal vein (CV) of tg(kdrl:mcherry-CAAX) ZFL. The red mCherry signal indicates endothelial cells. The yellow signal indicates colocalization. *B*: fluorescent signal of labeled test compounds in the tail region of 72-hpf ZFL. Experiments in the presence and absence of dextran sulfate (DXS; intravenous injection) 20 min before intravenous administration of the labeled compound are shown. *C*: signal intensity ratios between the CV and dorsal artery (DA) as determined for NBD-CsA, sulforhodamine 101 (Sulfo101), and fluo-cAMP. A ratio of >1 is indicative of accumulation in scavenger endothelial cells of the CV. –DXS, no inhibitor; +DXS, dextran sulfate (1 nL of 10 mg/mL). Values are means ± SD; *n* = 5. *P < 0.0001. Scale bars = 50 µm.

Analysis of the ZFL vasculature after injection of fluorescent labeled substrates of proximal tubule transporters revealed in some instances a punctuated staining pattern at the level of the dorsal CV (Figs. 4*A* and 5, *A* and *B*). Experiments in a transgenic fish line expressing mCherry in endothelial cells [tg(kdrl:mCherry-CAAX)] revealed colocalization of NBD-CsA with endothelial cells 1 hpi (Fig. 5*A*). Such signals were not observed in ZFL preinjected with dextran sulfate, a stabilin scavenger receptor inhibitor (Fig. 5*B*) (15). The same was also observed for Sulfo101 but not for fluo-cAMP. Quantitative analysis of signal intensity ratios between the CV and DA revealed a 1.5- to 2.5-fold increased accumulation at the level of the CV of Sulfo101 and NBD-CsA (Fig. 5*C*). By preinjecting ZFL with dextran sulfate, signal intensities led to a balanced CV-to-DA ratio of 1. Besides this, dextran sulfate had no effect on more hydrophilic compounds such as fluo-cAMP. These findings suggest inhibition of an endocytotic process in nonprofessional phagocytotic endothelia cells expressing the dextran sulfate-sensitive scavenger receptor stabilin-1/2. It is tempting to speculate that protein binding precedes stabilin receptor-mediated endocytosis since stabilin-1/2 is known to mediate blood clearance of macromolecules (15).

**Figure 6.**
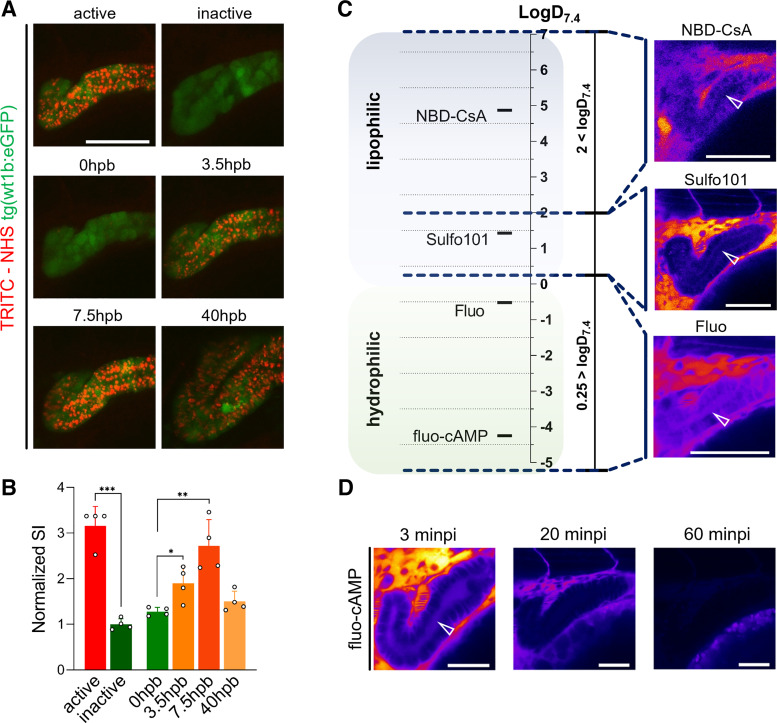
Endocytosis of protein-bound TRITC-NHS in the proximal convoluted tubule (PCT) and luminal secretion of hydro- and lipophilic fluorescent substrates. *A*: *top*: “active” indicates the PCT signal of a chemical reactive fluorescent compound (TRITC-NHS) 3 h postinjection (hpi); “inactive” indicates TRITC-NHS injected after inactivation by hydrolysis. *A*, *middle* and *bottom*: photobleaching of the PCT after injection of active TRITC-NHS and shown after 3.5, 7.5, and 40 h postbleaching (hpb). The green signal indicates 80-h postfertilization (hpf) tg(wt1b:eGFP) ZFL. Scale bar = 50 µm. *B*: quantitative assessment of TRITC-associated signals within the PCT (green) shown in *A*. Normalized SI is the PCT signal intensity compared with inactive TRITC-NHS. *C*: fluorescent substrates of proximal tubular drug transporters were categorized by lipophilicity (logD_7.4_ < 0.25 and logD_7.4_ > 2). Representative lateral projections of 72 hpf ZFL are shown to provide an alignment of hydrophilic/lipophilic classifications and the corresponding tubular signals. The white arrow indicates the tubular lumen. Scale bars = 50 µm. *D*: time-dependent depletion of the fluo-cAMP signal in the lumen of the PCT. Minpi, minutes postinjection. Scale bars = 30 µm. Values are means ± SD; *n* = 4. *P < 0.02; **P < 0.002; ***P < 0.0001.

To verify the hypothesis that endocytosis of protein-bound fluorescent small molecules gives rise to the observed punctuated staining patterns in ZFL, a chemically reactive fluorescent dye (TRITC-NHS), used as amine-reactive cross-linker for protein labeling, was intravenously injected. Indeed, a punctuated staining pattern within, for example, the PCT was observed (Fig. 6*A*). Qualitative and quantitative (Fig. 6*B*) evaluation of these signals revealed a threefold reduction of intensity when TRITS-NHS was hydrolyzed before injection. In a next step, the pronephros region was photobleached and the signal evolution was monitored over time. Signals reappeared after photobleaching being indicative of resequestration of fluorescent-labeled species. After photobleaching, signals reached a new maximum within 7.5 h, pointing to a very long half-life and thus persistence in the circulation of the labeled species.

We found that indirect assessment of renal clearance of fluorescent substrates based on their disappearance from the central blood compartment is more reliable than a measurement of their appearance within the PCT. Although direct transporter-mediated luminal secretion into the PCT of hydrophilic test compounds such as fluo-cAMP and fluorescein (logD_7.4_ < 0.25) could be visualized based on their appearance within the PCT, their lipophilic counterparts (i.e., Sulfo101 and NBD-CsA, logD_7.4_ > 0.25) could not be detected within the PCT but did accumulate within tubular epithelial cells (Fig. 6*C*). In these experiments, ZFL had to be analyzed within 10 minpi of 1 nL of 2 mM working solution due to rapid disappearance of fluorescent signals (Fig. 6*D*).

**Figure 7.**
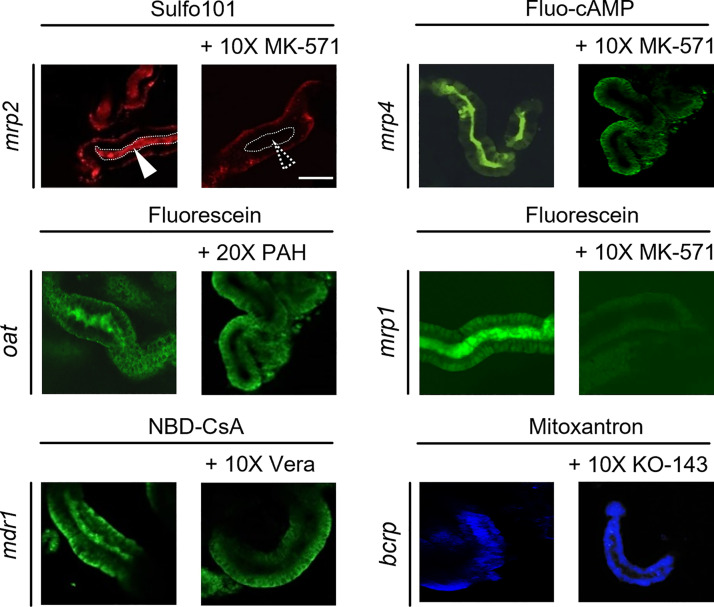
Excretion of fluorescent model substrates in isolated killifish proximal tubules in the presence or absence of specific inhibitors. Freshly isolated proximal tubules were incubated with fluorescent transporter substrates and analyzed by confocal microscopy. Renal secreted substrates were detected in the lumen of isolated proximal tubules. *Left*: no inhibitor. *Right*: incubations in the presence of fold excess of the indicated inhibitor. Signals within the luminal space (white arrows and dotted area) indicate tubular excretion of the transported substrate. Scale bar = 50 μm.

To allow for a direct assessment of proximal tubular secretion based on the appearance of fluorescent signals within the tubular lumen, isolated proximal tubules of killifish (*F. heteroclitus*) were used as a renal tubular transport model (Fig. 7). In contrast to the pronephros of the living ZFL, these isolated tubules are not patent but sealed due to partial collapse during the isolation procedure. Secreted fluorescent substrates could indeed be detected in the tubular lumen and excretion inhibited using corresponding transport inhibitors. Figure 7 shows a qualitative assessment by confocal fluorescence microscopy of accumulated transporter substrates (1 µM) within the proximal tubular lumen in the presence and absence of a 10- to 20-fold excess of the correlating transport inhibitor. Image analysis allowed for a statistical assessment of specific transporter inhibition. The remaining activity in the presence of inhibitor for the ABC transporters was 65 ± 7% for mrp2 (abcc2) (substrate: Sulfo 101, inhibitor: MK571), 55 ± 8% for mrp4 (abcc4) (substrate: fluo-cAMP, inhibitor: MK571), 45 ± 8% for mdr1 (abcb1) (substrate: NBD-CsA, inhibitor: verapamil), and 60 ± 9% for bcrp (abcg2) (substrate: mitoxantrone, inhibitor: KO-143). For the SLC transporter oat (slc22), excretion of fluorescein was reduced to 72 ± 12% in the presence of *p*-aminohippurate and to 11 ± 15% in the presence of MK-571. All values are presented as means ± SD, *n* = 12 and showed a level of significance of *P* < 0.05 compared with control (no inhibitor).

## DISCUSSION

Anatomic structures of the developing pronephros in teleosts show high similarity to corresponding structures in mammals, including humans (12, 45). This notion is supported by the observation that, for example, transcription factors responsible for patterning the developing kidney in zebrafish are evolutionary conserved in mammals (2, 45, 46). With respect to renal functionality, the question arises if ZFL can be used as a vertebrate screening model to study renal processing of pharmaceutics. It was therefore the aim of this study to evaluate to which degree the ZFL pronephros functionality reflects the situation in higher vertebrates, including humans.

To control chemical exposure within ZFL, all test compounds were injected intravenously into the duct of Cuvier, resulting in a bioavailability of 100%. By this approach, potential interference by intestinal metabolism can be as well excluded. Of note, alternative clearance mechanisms via the liver are not considered to take place since liver associated xenobiotic metabolism is not operational by 5 dpf (47, 48). Injection volumes in these experiments were 1–2 nL, which corresponds to an estimated 2% of the total blood volume of a 72-hpf ZFL. Injected solutions did contain a maximum of 15% DMSO. This procedure was well tolerated by the ZFL as demonstrated by monitoring of heart beat and viability during the duration of control experiments (i.e., injection of DMSO-PBS followed by 2-h monitoring). There were no statistically significant changes in heart beat (*n* = 16 ZFL) and no apparent signs of toxicity.

Of note, blood concentrations of circulating fluorophores were measured within the DA. Signals in the CV are indicative of cellular uptake by nonprofessional phagocytic cells. This process is mediated by endothelial scavenger receptors (e.g., stabilin-1/2), as shown in Fig. 5.

In the first series of experiments, we could confirm functionality of the ZFL glomerulus at 96 hpf by the determination of a *D*_H_ threshold for glomerular filtration in the range of 4.4–7.6 nm. This is in line with studies in rats (49, 50) that demonstrated that the majority of macromolecules are retained within the glomerulus by small pores of a radius 4–5 nm or negatively charged slit diaphragms of radius 6 nm. Thus, the passage of proteins such as, for example, albumin (human serum albumin: 69 kDa, 7- to 10-nm diameter) (51) is restricted across these pores (52). The contribution of the glomerular basement membrane and its morphology toward glomerular filtration has been previously discussed (53, 54).

Although ZFL, before reaching 96 hpf, are reported to have size-dependent glomerular filtration (55), there are reports indicating that slit diaphragm formation is not yet completed at this development stage (52). Therefore, we used in this study 96-hpf ZFL. We assume that at this stage podocyte foot processes, endothelial cell fenestrations, and slit diaphragms are mature and fully functional and therefore justify the present use of ZFL as an in vivo vertebrate model to study renal function (24, 35).

Our experiments were carried out using fluorescent labeled PEG or dextran of different molecular weight, whose purity and hydrodynamic radii were determined by fluorescence correlation spectroscopy. Dextran and PEG are frequently used as pharmaceutical excipients (56). Covalently bound, PEG sterically stabilizes macromolecules and drug-containing particles (56). In the systemic circulation, PEG increases the half-life of these molecules. A few studies have been done describing characteristics of renal clearance based on PEG length (57). Here, our study revealed that rather the *D*_H_ of PEG and dextran polymers determines renal filtration properties instead of molecular weights, i.e., 40-kDa PEG is not filtrated, whereas 40-kDa dextran is rapidly cleared. In ZFL, these results confirm previous reports on size-selective glomerular filtration in 72-hpf ZFL (55, 58) and 96-hpf ZFL [e.g., 10- vs. 500-kDa dextran polymer (59)], albeit we are the first who determined a clear cutoff value.

Distal tubular reabsorption was assessed based on receptor-mediated transport of FA/vitamin B_9_ via folr1 (ZLF analog of the human FOLR1 receptor) (3). FOLR1 is expressed on the apical side of PCT cells (60) and facilitates transcytosis (61). However, folr1 is also conserved in a wide range of vertebrates. Recently, homolog mRNA expression of human FOLR1 was detected in zebrafish throughout embryogenesis in distal tubules and showed high structural homology of the FA-binding site with vertebrates and humans. In particular, FA-binding site 1, consisting of five amino acids, is conserved in cows, mice, and rats (3). In our study, we used a fluorescent labeled FA-PEG polymer with an experimentally determined *D*_H_ of 1.1 nm, which is in alignment with literature reports of similar PEG polymers (62). The fluorescently labeled FA analog was retained in the ZFL circulation but was rapidly renal excreted in presence of an excess of nonlabeled FA. Our inhibition experiments thus demonstrate specific and receptor-mediated distal tubular reuptake of FA by a FA receptor-mediated process. It should be noted that the tubular system in the living ZFL is patent, i.e., urine is rapidly expelled to the surrounding media by the continuous action of cilia lining the inner surface of the tubules (63). It should be noted that FA-PEG2000-FITC is present in the distal tubular lumen but hardly visible in the surrounding tubular epithelial cells. We have observed a similar phenomenon earlier in a transendothelial transport study using a fluorescent-labeled IgG monoclonal antibody directed against the rodent transferrin receptor (64). In these experiments, transcellular receptor-mediated transport (i.e., transcytosis) was demonstrated to be a highly efficient and fast process resulting in very low steady-state concentrations of the transported IgG within endothelial cells.

Transporter-mediated proximal tubular excretion of fluorescent labeled xenobiotics was studied based on their appearance within the PCT or, alternatively, their disappearance from the central blood compartment. Criteria for the selection of the used fluorescent substrates and nonfluorescent inhibitors as well as information on their selectivity and use by other authors are shown in Table 2. The studied ABC transporters include abcb4, mrp1 (abcc1), mrp2 (abcc2), and mrp4 (abcc4). mRNA expression levels in ZFL determined between 24 and 120 hpf point to homolog expression of these transporters in teleosts and mammals, including humans, in various organs and proximal tubules (6–8). The abcb4 transporter has been described as a homolog of human p-glycoprotein/MDR1 (9). In this study, we could demonstrate that these transporters are fully functional in ZFL at 72 hpf and mediate active secretion into the PCT of their respective substrates. SLC [i.e., oat (slc22)] transport functionality was confirmed using negatively charged Fluo as a substrate (34, 65). In addition, we could show in this study that fluorescein was transported by an mrp transporter. This finding suggests functional expression of mrp1 in zebrafish since, first, fluorescein is a substrate of human MRP1 (ABCC1) (66) and, second, mrp1 expression in zebrafish has been previously demonstrated by genetic analysis (7). Specificity of transport was demonstrated in these experiments using combinations of fluorescent transporter substrates and their respective inhibitors. Again, the appearance of fluorescent signals within the tubular lumen was a less reliable measure compared with disappearance of transporter substrates from the central blood compartment, recorded based on fluorescent signals present in the DA. This underlines that the pronephros of ZFL is an open fluid compartment and that clearance of drugs is a rapid process. The higher luminal accumulation of hydrophilic substances can be explained by the fact that hydrophilic compounds experience both saturable active as well as dose-linear passive (glomerular filtration) clearance, whereas most lipophilic compounds are only transported actively (4). Furthermore, some lipophilic compounds, such as NBD-CsA, did not reveal any luminal signals but showed an association with renal epithelial cells instead. Consequently, hydrophilic compounds are cleared faster and show higher transient signals in the tubular lumen.

Although the human homolog p-glycoprotein/MDR1 (ABCB1) gene is absent in ZFL, active secretion of lipophilic, uncharged, or moderately basic substrates in ZFL can be compensated by an abcb4 transporter (2, 9, 67). Furthermore, and to the best of our knowledge, the results of our study suggest, for the first time, functional expression of mrp1 (abcc1) in teleosts, i.e., zebrafish and killifish.

We cannot exclude that expression of drug transporters outside of the pronephros may have an impact on the indirect measurements of fluorescent substrates within the blood compartment. This can potentially lead to the accumulation of fluorescent signals in specific organs and tissues such as the brain or developing liver. However, we have not observed such effects (e.g., Fig. 4). This can be explained by the fact that drug transporters studied in the present work have a protective function preventing cellular accumulation. Of note, stabilin-mediated endocytosis by endothelia of the CV leads to a punctuated staining pattern. This latter observation has prompted us to study elimination of circulating compounds by pathways others than renal excretion in greater detail.

When comparing tail images from our transporter experiments, a punctuated staining pattern after injection of NBD-CSA and Sulfo101 was observed within the CV. Experiments with 72-hpf tg(kdrl:mCherry-CAAX) ZFL expressing mCherry in endothelial cells revealed a colocalization of fluorescent signals. This observation is indicative of cellular uptake of fluorescent molecules by scavenger endothelial cells located in the CV. Such endothelial cells with a scavenging function can be found in various organs in teleost fish, sharks, and lampreys (68). In mammals, they predominantly line the liver sinusoids. In a previous study (15), we could demonstrate stabilin-2-dependent scavenging of lipid nanoparticles in the CV region of ZFL. In this as well as the present study, stabilin-2-mediated clearance could be selectively blocked by preinjection of dextran sulfate. Dextran sulfate is an inhibitor of stabilin-2 and related scavenger receptors. Since stabilin-2 mediates cellular uptake of negatively charged macromolecules or nanoparticles by a clathrin-coated pit pathway (69), it is reasonable to assume that in our experiments not the free fluorescent small molecule was recognized but a protein-associated conjugate thereof. Indeed, cyclosporine analogs are characterized by a very high protein binding of >98% (70). Sulfo101 or Texas red is used as an astrocyte-specific marker (71). Its hydrolysis product is a water-soluble sulfonic acid derivative, which shows reduced adsorption to proteins. This is in line with the observation that preincubation of Sulfo101 in serum or buffer reduces the appearance of the punctuated staining pattern in zebrafish. These experiments suggest that protein adducts accumulate within endothelial cells of the CV by a stabilin-mediated endocytotic process.

Of interest, chemically reactive TRITC-NHS (amine-reactivity via *N*-hydroxysuccinimide) showed an additional punctuated staining of the PCT. This finding is in line with previous reports of PCT endocytosis of smaller 10-kDa dextran-FITC conjugates (72, 73) or endocytosis and lysosomal processing of a red fluorescent protein consisting of the monomeric vitamin D-binding protein (1/2vdp-mCherry) (74). Again, the punctuated staining pattern was only visible using the chemically reactive fluorescent marker but not when using its hydrolyzed counterpart. This and photobleaching experiments suggest that long circulating protein adducts were present in the circulation up to 40 h postbleaching, leading to continuous endocytosis and cellular accumulation at the level of the PCT. It remains to be elucidated by which mechanism the protein adducts are reabsorbed from the tubular lumen.

To confirm the conservation of transport functions in an additional teleost species, freshly isolated and sealed tubuli of killifish (*F. heteroclitus*) were used as a complementary ex vivo transport model (12). These tubuli are characterized by collapsed and thus sealed ends. Therefore, accumulation of transporter substrates within a closed tubular lumen can be monitored (18, 34). Indeed, using the same representative inhibitors and fluorescent substrate pairs, our results in ZFL could be confirmed. This supports that tubular transporters are highly conserved in teleosts.

### Perspectives and Significance

The ZFL is an attractive in vivo vertebrate model that is extensively used, for instance, in toxicological, pharmacological, and nanomedicine research (16, 58). Our study revealed that ZFL at 96 hpf have a fully functional pronephros. Glomerular filtration is characterized by a cutoff similar to that of higher vertebrates. Small-molecule substrates of ABC and SLC transporters are actively secreted at the level of the proximal tubule. Receptor-mediated endocytosis by the FA receptor could be demonstrated for the distal tubule. The here-proposed protocol uses intravenous injections (allowing for a precise dosing and defined exposure of the ZFL) in combination with fluorescent reference compounds to study renal function in 3- to 4-dpf larvae. If transport experiments are carried out using calibrated amounts of transport substrates dose-dependent kinetic effects can be monitored. During such experiments, additional information can be obtained with respect to the tolerability, circulation behavior, extravasation, cellular interaction, and tissue accumulation of test compounds in vivo. The ZFL model provides a higher throughput compared with alternative screening models in vertebrates. It can therefore be considered to be a cost-effective and attractive translational tool to bridge the gap between cell culture-based test systems and pharmacokinetic experiments in higher vertebrates such as rodents. We propose that this model can be used as a screening model to identify interactions of unknown test compounds with renal transport based on their interactions with coinjected fluorescent markers.

## GRANTS

J.S.B. was supported by the Stiftung zur Förderung des pharmazeutischen Nachwuchses in Basel. J.S.B., G.F., and J.H. were supported by Maine INBRE Grant GM103423, Salisbury Cove Research Foundation, and Ulric Dahlgren Fund.

## DISCLOSURES

No conflicts of interest, financial or otherwise, are declared by the authors.

## AUTHOR CONTRIBUTIONS

J.S.B., A.P., C.L.A., and J.H. conceived and designed research; J.S.B., A.P., C.L.A., G.F., and J.H. performed experiments; J.S.B., A.P., C.L.A., and J.H. analyzed data; J.S.B., A.P., C.L.A., G.F., and J.H. interpreted results of experiments; J.S.B., C.L.A., and G.F. prepared figures; J.S.B., C.L.A., and J.H. drafted manuscript; J.S.B., A.P., C.L.A., G.F., and J.H. edited and revised manuscript; J.S.B., A.P., C.L.A., G.F., and J.H. approved final version of manuscript.
